# Cross-sectional and longitudinal factors influencing physical activity of 65 to 75-year-olds: a pan European cohort study based on the survey of health, ageing and retirement in Europe (SHARE)

**DOI:** 10.1186/s12877-018-0781-8

**Published:** 2018-04-16

**Authors:** Lena Lübs, Jenny Peplies, Carina Drell, Karin Bammann

**Affiliations:** Institute for Public Health and Nursing Sciences (IPP), University of Bremen, Bremen, Germany

**Keywords:** Physical activity, Older adults, SHARE, Ageing, Europe, Cohort study, Risk factors

## Abstract

**Background:**

The promotion of physical activity (PA) plays a major role for healthy ageing even in older age. There is a lack of cross-sectional and longitudinal studies explicitly dealing with barriers and drivers to PA in older adults. Therefore the aims of this study are a) to determine the prevalence of insufficient physical activity (IPA) in 65 to 75-year-olds in Europe and to identify factors associated with IPA in cross-section and b) to identify longitudinal risk factors for IPA in prior active persons.

**Methods:**

This study is using data of the Survey of Health, Ageing and Retirement in Europe (SHARE). SHARE is a cross-national panel database including individual data of the non-institutionalised population aged 50+ from 27 European countries. For the present paper, we included a cohort that participated in all first four waves of SHARE (2004–2011) aged 65-to-75–years at wave four (male *n* = 1761, female *n* = 2085) from 10 European countries. To identify cross-sectional and longitudinal associations, we calculated prevalence odds ratios and hazard ratios with 95% confidence intervals.

**Results:**

The prevalence of IPA in 65–75-year-olds varied widely between countries, ranging from 55.4% to 83.3% in women and from 46.6% to 73.7% in men. IPA was associated with several intrapersonal factors and strength of association was similar for men and women for almost all investigated factors. Statistically significant associated with IPA were socioeconomic factors as low educational level (own and parental) and financial difficulties (male: POR: 1.60: 95%-CI: 1.26–2.03; female: POR: 1.58; 95%-CI: 1.26–1.97) and health-related factors as e.g. number of chronic diseases (male: POR: 1.34: 95%-CI: 1.23–1.45; female: POR: 1.31; 95%-CI: 1.21–1.42). Interpersonal only the size of social network was associated with IPA (male and female: POR: 0.88, 95%-CI: 0.81–0.95). Longitudinally in a fully adjusted model, only grip strength (HR: 0.99; CI-95%: 0.98–0.99) and BMI (HR: 1.02; CI-95%: 1.00–1.04) were statistically significant risk factors for IPA.

**Conclusions:**

PA promotion programs for older adults should incorporate the heterogeneity of health status and physical condition that can typically occur in this age group.

**Electronic supplementary material:**

The online version of this article (10.1186/s12877-018-0781-8) contains supplementary material, which is available to authorized users.

## Background

Demographic change is a challenging development for the social systems of all member states in the European Union (EU) [1, 2]. The feared impact of demographic change could be mitigated by healthy ageing, as older adults in good health remain longer at work or can play an active role in society through volunteer activities [3]. Physical activity (PA) is essential for the skeletal, muscular- and digestive systems and also for circulation [4]. In this context, PA plays a major role as it can increase life expectancy, daily living skills, overall well-being and quality of life [5–8]. An estimated amount of three million premature deaths can be attributed to lack of PA, which could have been avoided through prevention and health promotion [5, 6, 9, 10].

In the EU, the main causes of death are diseases of the cardiovascular system for which lack of PA is one of the major risk factors [5, 6, 9, 11, 12]. Thus, lack of PA is related to one of the major cost factors in the EU health systems [4, 6]. Sufficiently active people have previously shown to carry a lower risk of poor health or development of chronic diseases in old age [5, 13]. Even in older adults, regular PA can still improve mental and physical health and positively affect the general ageing process [4, 5].

The WHO recommends at least 2.5 h/week of moderate or 75 min/week of vigorous PA [13]. In Europe, 35% of adults are considered as physically inactive and this proportion increases with age to 45% of the 60 + −year-olds [4]. Prevalence of PA is different in the European countries, people of southern Europe are less active compared to other areas [14]. In average, women are less active than men [15].

Particularly with regard to increasing life expectancy in the EU, PA opportunities in everyday life of older adults require special attention in terms of promoting PA. The transition to retirement is a promising starting point for interventions promoting an active lifestyle, because people tend to establish new routines and give up previous ones. For example occupational PA opportunities, such as PA at work or active transport to workplace, are no longer relevant. Van Dyck et al. showed that this opportunity not only lasts during the actual transition to retirement, but also during the first years of retirement [16]. In order to pursue a holistic approach to health promotion, variables of all levels should be included in accordance to Bronfenbrenners ecological systems theory [17, 18].

Based on the three functional domains proposed by Livneh [19] and on the classification Bauman et al. (2012) used, determinants of PA can be categorised into intrapersonal, interpersonal and extrapersonal factors. Intrapersonal factors comprise factors related to a person’s mind or self, such as health and psychological well-being. Intrapersonal factors that are negatively associated with PA are age and female gender [20–23], poor health status [20, 22, 24, 25], perceived frailty [22, 23, 26], low socio-economic status [20, 22], low parental socio-economic position [27, 28] and high Body Mass Index (BMI) [20, 22, 23]. Positively associated are sufficient PA during the life course [20, 22, 23, 26], as well as self-efficacy and the belief in the benefits of PA [20, 22–24, 26].

Interpersonal factors refer to family and marital life as well as peer and social relations. It has been shown that social support of family members, friends, sports partners and trainers are important positive factors for PA [20, 22, 26, 29–32]. Additionally, social contact and a social network in neighbourhood enhance PA in older adults [22, 33, 34]. McNeil et al. stated that social networks can influence PA positively by providing social support and establishing social norms that enable health-promoting behaviours [35].

Extrapersonal factors are community-based and therefore beyond the personal or individual dimension, such as policies, physical and social environments. In this regard, economic conditions and societal norms are important determinants for PA in adults [20, 22]. Likewise, built environment and walkability of a neighbourhood can influence PA in older adults negatively and also positively [31, 36–39]. Moreover, a familiar neighbourhood [40], security in the neighbourhood in terms of traffic and crime as well as access to a PA-promoting infrastructure affect PA positively [31, 39, 40]. In this study, no extra-personal factors are included because the SHARE dataset only collected the resident of the participants and no extra-personal factors at community level.

PA-influencing factors vary between age groups. Therefore, transferability of results from other age groups is limited. There is a lack of studies explicitly dealing with factors influencing PA in older adults in Europe. This especially holds for high-quality longitudinal studies [41, 42] and for studies using objective PA measurements [20]. In this paper, we re-analyse the data of the Survey of Health, Ageing and Retirement in Europe (SHARE). SHARE is a longitudinal study that includes several countries using a common standard and thus allows inter-country comparisons. Although SHARE does not use objective PA measurement, but self-reported PA, the broad range of factors, the high degree of standardisation and the longitudinal nature of the data makes it a valuable resource for research.

The aims of this study are a) to determine the prevalence IPA in 65 to 75-year-olds in Europe and to identify factors associated with IPA in this age group and b) to identify longitudinal risk factors for IPA in prior active persons using the data of the Survey of Health, Ageing and Retirement in Europe (SHARE).

## Methods

### Study design and population

This study is conducted using data of the interdisciplinary panel Survey of Health, Ageing and Retirement in Europe (SHARE), which is performed in 19 countries of the European Union and Israel. The aim of the SHARE-project is to provide an overall picture of ageing in Europe and it gathered data on health, socio-economic status and social as well as family networks [43]. In SHARE, non-institutionalised people aged 50+ underwent a short physical examination and were interviewed with computer assisted personal interviews (CAPI). Wave one took place in 2004/2005, wave two in 2006/2007, wave three in 2008/2009 and wave four in 2010/2011. SHARE has data of more than 60,000 individuals, the response rate in wave one was around 62%, in waves two, three and four 73%, 77% und 56% [43]. The ten countries participating in all first four waves of SHARE were Belgium, Denmark, Germany, the Netherlands, France, Austria, Italy, Switzerland, Sweden and Spain. For further methodological details of SHARE see Börsch-Supan et al. (2013) [43]. This study is following the Strengthening the Reporting of Observational Studies in Epidemiology (STROBE) Statement [44]. An additional file shows this in more detail [see Additional file 1]. Inclusion criteria for this study were participation in all four waves of SHARE and belonging to the age group of 65-to-75–year-olds at the time of wave four. This resulted in a sample size of *n* = 3846 (male *n* = 1761, female *n* = 2085).

### Outcome and country of residence

Physical activity was assessed based on the following two questions: ‘How often do you engage in vigorous physical activity, such as sports, heavy housework, or a job that involves physical labour?’ and ‘How often do you engage in activities that require a low or moderate level of energy such as gardening, cleaning the car or doing a walk?’. Given possible answers were ‘More than once a week’, ‘once a week’, ‘one to three times a month’ and ‘hardly ever’ or ‘never’. Participants, who stated to engage in vigorous physical activity once a week or less were defined as *insufficient physical activity (IPA)* (wave 4: *n* = 2582; 67.2%). Participants engaging in vigorous physical activity more than once a week are categorized as sufficient PA and serve as control group. The WHO used IPA as measurement and defined it as less than 150 min of moderate physical activity or less than 75 min of vigorous physical activity per week [45]. The variable *country* is defined as the country of residence of the survey participants.

### Covariables – Intrapersonal factors

*Age* was calculated from birth month and year of the participants and time of the interview in wave four. The exact age in months was divided by 12 to generate age in years. *Level of education* was classified by the International Standard Classification of Education (ISCED) [46]. For *parental education,* the number of books present in the household that the participants lived in at the age of ten was used [47].The variable was dichotomised. Zero to eleven books were classified as ‘low parental education’ and more than eleven books as ‘higher parental education’. The *financial situation* was assessed with the question whether a household has trouble or not to make ends meet with the available monthly income. Four answer categories were used: ‘with great difficulty’, ‘with some difficulty’, ‘fairly easily’ and ‘easily’. The variable was dichotomised. All participants answering ‘with great difficulty’ and ‘with some difficulty’ were graded as ‘without difficulty’ and ‘fairly easily’ and ‘easily’ as without difficulties. The *number of chronic diseases* was based on a multiple answer question. The numeric variable was truncated at a number of three chronic diseases. *Depression* was examined with the EURO-D symptom scale, an index consisting of twelve items: depressed mood, pessimism, suicidality, guilt, sleep, interest, irritability, appetite, fatigue, concentration, enjoyment and tearfulness. The scale ranges from zero ‘not depressed’ to twelve ‘very depressed’. The variable was dichotomised according to Dewey and Prince into ‘no depression’ (0–3) and ‘depression’ (4–12) [48]. *Hospitalization in the last twelve months* shows whether participants were in a medical, surgical, psychiatric or any other specialized hospital overnight during the last twelve months before the interview. Two *grip strength* measurements on each hand were recorded using a dynamometer (Smedley, S Dynamometer, TTM, Tokyo, 100 kg). The variable shows the maximum grip strength of all four measurements. To describe *limitations in activities of daily living* (ADL) the activities of daily living index (ADLI) was used [49]. The ADLI is the sum of the five tasks dressing, bathing or showering, eating, cutting up food, walking across a room and getting into or out of bed. The higher the index the more difficulties exist with these activities. The variable was dichotomised. All participants with the index value zero were graded as ‘without limitations’ and with the index value one to five as with limitations. *Body Mass Index* (BMI) is based on self-reported values and is calculated as: *BMI = weight in kilogram (kg) / (height in meter (m))* ^*2*^*.*

### Covariables – Interpersonal factors

To determine *marital status* the existing response categories ‘married and living together with spouse’, ‘married, living separated from spouse’ and ‘registered partnership’ were summarised in the category ‘married and registered partnership’. The dichotomous variable *partner in household* indicates whether a participant was living together with his partner in a household. To measure the *size of family network*, a score was calculated from the *number of children alive*, the *number of parents alive* and the *number of siblings alive*. The numeric variable *number of children* was truncated at a number of four children. For four or more children, four children were included into the score. A high score value indicates a large family network. From wave four of SHARE on, information on up to seven individuals with whom respondents discussed important things most often during the 12 months before the interview is available. The *size of social network* may include family members, friends, neighbours and others and was truncated at three network partners. The dichotomous variable p*erceived social support* shows whether the respondent had received instrumental social support within the last 12 month. The *perceived quality of social network* was assessed on a scale ranging from ‘0’ (completely dissatisfied) to ‘10’ (completely satisfied), including respondents who reported to have no social network.

### Statistical analyses

To identify cross-sectional associations of intra- and interpersonal factors with IPA in wave four, prevalence odds ratios (POR) and 95%-confidence intervals (95%-CI) were calculated using binary logistic regression models.

To analyse the longitudinal association over time, we calculated hazard rations (HR) with 95% confidence intervals (95%-CI) using Cox regression models in the group of sufficient PA in wave 1. IPA at wave 4 served as the outcome. To account for heterogeneity between the observed countries, we included country as random effect in our models. We omitted stratification by sex for intrapersonal factors, because in the cross-sectional analyses the effects were similar for men and women. For interpersonal factors this was not the case, therefore we stratified the analyses by sex. Model building was based on the Wald statistic. But previously we calculated all other inclusion methods (backward, forward and expected inclusion) with the result that the method did not affect the result of regression after adjustment.

All analyses were performed using the statistical analysis software IBM SPSS statistics for Windows, version 20.0 (IBM Corp. Released 2011. Armonk, NY).

## Results

A high proportion of our study group completed an upper secondary education or higher (women: 45.5%; men: 58.4%, Table 1). Most participants were married (female: 68.5%; male: 84.4%). A considerably higher proportion of women than men was widowed (female: 18.2%; male: 5.2%) and lived in single households (female: 29.2%; male: 13.1%). The majority of the sample had at least one child (female: 92.1%; male: 90.9%).

**Table 1:** Description of the study sample

	Male (*n* = 1761)	Female (*n* = 2085)
Age at wave 4: Mean (SD^1^)	69.6 (3.15)	69.7 (3.13)
Country		
Sweden	206 (11.7%)	256 (12.3%)
Denmark	138 (7.8%)	134 (6.4%)
Netherlands	193 (11.0%)	216 (10.4%)
Germany	202 (11.5%)	196 (9.4%)
Belgium	279 (15.8%)	345 (16.5%)
Austria	95 (5.4%)	122 (5.9%)
Switzerland	90 (5.1%)	105 (5.0%)
France	158 (9.0%)	230 (11.0%)
Italy	266 (15.1%)	311 (14.9%)
Spain	134 (7.6%)	170 (8.2%)
Educational level		
Primary education	442 (25.1%)	676 (32.4%)
Lower secondary education	279 (15.8%)	446 (21.4%)
Upper secondary education	517 (29.4%)	510 (24.5%)
Tertiary education	511 (29.0%)	439 (21.0%)
Marital Status wave 4		
Never married	87 (4.9%)	106 (5.1%)
Married	1486 (84.4%)	2029 (68.5%)
Divorced	94 (5.3%)	166 (8.0%)
Widowed	92 (5.2%)	380 (18.2%)
Size of household wave 4		
1-person household	231 (13.1%)	609 (29.2%)
2-person household	1277 (72.5%)	1292 (62.0%)
More than 2-person household	253 (14.4%)	184 (8.8%)
Lives together with partner in household wave 4		
Yes	1502 (85.3%)	1387 (66.5%)
No	259 (14.7%)	698 (33.5%)
Number of children		
None	144 (8.2%)	152 (7.3%)
1–2 children	986 (55.9%)	1130 (54.2%)
3 and more children	615 (35.0%)	789 (37.9%)

^1^SD = standard deviation

Within the 65–75 year-olds, 67.2% reported IPA with women being less active than men and an increasing trend by age group in both genders (Table 2). At baseline, prevalence of IPA in the different countries ranged from 42.1% (Germany) to 63.5% (Italy) in men and from 48.5% (Denmark) to 75.9% (Belgium) in women. The steepest increase in IPA within seven years were seen in Danish women (wave 1: 48.5%; wave 4: 66.2%) and in German men (wave 1: 42.1%; wave 4: 55.2%).

**Table 2:** Longitudinal development of prevalence rates (PR) and 95%-confidence interval (95%-CI) of insufficient physical activity (IPA)

Male (*n* = 1760)				Female (*n* = 2085)			
	Wave 1 baseline* PR (95%-CI)	Wave 2 2 years later PR (95%-CI)	Wave 4 7 years later PR (95%-CI)		Wave 1 baseline* PR (95%-CI)	Wave 2 2 years later PR (95%-CI)	Wave 4 7 years later PR (95%-CI)
Countries				Countries			
Sweden (*n* = 206)	52.9% (46.1–59.7)	53.7% (46.9–60.5)	46.6% (39.8–53.4)	*Sweden (n = 256)*	61.7% (55.7–67.7)	58.8% (52.8–64.8)	64.1% (58.2–70.0)
Denmark (*n* = 138)	47.1% (38.7–55.5)	55.1% (46.8–63.4)	59.1% (52.4–65.8)	*Denmark* (*n* = 134)	48.5%** (40.0–57.0)	64.1% (55.9–72.3)	66.2% (58.2–74.2)
Netherlands (*n* = 193)	49.2% (42.1–56.3)	48.4% (41.3–55.5)	52.4% (45.3–59.5)	*Netherlands* (*n* = 216)	55.6% (49.0–62.2)	47.9% (41.2–54.6)	59.5% (52.9–66.1)
Germany (*n* = 202)	42.1%** (35.3–48.9)	55.4% (48.5–62.3)	55.2%** (48.3–62.1)	*Germany* (*n* = 196)	49.0%** (42.0–56.0)	60.2% (53.3–67.1)	55.4% (48.4–62.4)
Belgium (*n* = 279)	60.9% (55.2–66.6)	59.5% (53.7–65.3)	63.1% (57.4–68.8)	*Belgium* (*n* = 345)	75.9% (71.4–80.4)	76.7% (72.2–81.2)	80.8% (76.6–85.0)
Austria (*n* = 95)	63.2% (53.5–72.9)	61.1% (51.2–71.0)	58.5%** (48.5–68.5)	*Austria* (*n* = 122)	73.0% (65.1–80.9)	72.7% (64.8–80.6)	81.8%** (74.9–88.7)
Switzerland (*n* = 90)	42.2% (31.9–52.5)	42.2% (31.9–52.5)	53.3% (42.9–63.7)	*Switzerland* (*n* = 105)	54.3% (44.7–63.9)	52.4% (42.8–62.0)	67.6% (58.6–76.6)
France (*n* = 158)	59.2% (51.5–66.9)	66.2% (58.8–73.6)	69.4% (62.2–76.6)	*France* (*n* = 230)	77.8% (72.4–83.2)	80.3% (75.1–85.5)	80.0% (74.8–85.2)
Italy (*n* = 266)	63.5% (57.7–69.3)	66.5% (60.8–72.2)	73.2% (67.9–78.5)	*Italy* (*n* = 311)	71.4% (66.4–76.4)	74.3% (69.4–79.2)	83.3% (79.1–87.5)
Spain (*n* = 134)	61.2% (52.9–69.5)	61.7% (53.4–70.0)	73.7% (66.2–81.2)	*Spain* (*n* = 170)	68.0% (61.0–75.0)	66.3% (59.2–73.4)	82.1% (76.3–87.9)
Total (*n* = 1761)	54.9% (52.6–57.2)	57.8% (55.5–60.1)	60.9% (58.6–63.2)	*Total* (n = 2085)	65.4% (63.4–67.4)	66.9% (64.9–68.9)	73.0% (71.1–74.9)

*Age at baseline 58–68 years; ** Deviation of more than 5 percentage points from prevalence rates of all participants aged 65–75 years at wave 4 (see Additional file 1)

All **intrapersonal factors** showed a significant cross-sectional association with IPA in both sexes. Strength of association was similar for men and women for almost all investigated factors. Protective factors for IPA were higher education (male: POR: 0.57: 95%-Ci: 0.46–0.70; female: POR: 0.51; 95%-CI: 0.41–0.64), education of the parents (male: POR: 0.57: 95%-Ci: 0.47–0.70; female: POR: 0.53; 95%-CI: 0.44–0.66) and grip strength in kg (male: POR: 0.95: 95%-Ci: 0.94–0.96; female: POR: 0.96; 95%-CI: 0.94–0.97). Factors increasing the risk in older adults for IPA are age (male: POR: 1.04: 95%-Ci: 1.01–1.07; female: POR: 1.05; 95%-CI: 1.02–1.09), a difficult financial situation of the household (male: POR: 1.60: 95%-Ci: 1.26–2.03; female: POR: 1.58; 95%-CI: 1.26–1.97), a higher BMI (male: POR: 1.05: 95%-Ci: 1.03–1.08; female: POR: 1.05; 95%-CI: 1.03–1.08), number of chronic diseases (male: POR: 1.34: 95%-Ci: 1.23–1.45; female: POR: 1.31; 95%-CI: 1.21–1.42), depression (male: POR: 1.24: 95%-Ci: 1.13–1.35; female: POR: 1.17; 95%-CI: 1.09–1.25) and limitations in the activities of daily living (male: POR: 2.88: 95%-Ci: 1.88–4.39; female: POR: 2.45; 95%-CI: 1.64–3.68). The greatest gender-related difference was seen for hospitalisation within the last 12 months, that was a risk factor for IPA in both sexes, but stronger in women (POR: 2.18; 95%-CI: 1.59–2.98) than in men (POR: 1.36; 95%-CI: 1.05–1.77).

Very few of the investigated **interpersonal factors** had a statistically significant association with the prevalence of IPA (Table 3). A significant influence on both sexes was shown by the size of social network (POR: 0.88, 95%-CI: 0.81–0.95). All other interpersonal factors differed between sexes. In women but not in men, divorce was a protective factor for IPA (POR: 0.65; 95%-CI: 0.46–0.91). Single and widowed men had a higher risk of IPA (never married: POR: 1.92: 95%-Ci: 1.17–3.15; widowed: POR: 2.07; 95%-CI: 1.27–3.39). In men, a lower risk of IPA was seen for living together with a partner in household (POR: 0.59; 95%-CI: 0.44–0.79), the number of grandchildren (POR: 0.96; 95%-CI: 0.94–0.98), the size of family network without partner (POR: 0.94; 95%-CI: 0.90–0.99) and the perceived social support (POR: 1.44, 95%-CI: 1.01–2.07). None of these variables had a cross-sectional association with IPA in women.

**Table 3:** Cross-sectional association of intra- and interpersonal factors for insufficient physical activity (IPA) in 65–75-years-olds

	Male (*n* = 1761)		Female (*n* = 2085)	
	Prevalence odds ratio	95%-confidence interval	Prevalence odds ratio	95%-confidence interval
Intrapersonal factors				
Age in years	1.04**	1.01–1.07	1.05***	1.02–1.09
Higher Education (yes/no)	0.57***	0.46–0.70	0.51***	0.41–0.64
Books at the age of 10 years (yes/no)	0.57***	0.47–0.70	0.53***	0.44–0.66
Difficult financial situation of household (yes/no)	1.60***	1.26–2.03	1.58***	1.26–1.97
Number of chronic diseases	1.34***	1.23–1.45	1.31***	1.21–1.42
Depression (yes/no)	1.24***	1.13–1.35	1.17***	1.09–1.25
Hospitalization in the last 12 months (yes/no)	1.36*	1.05–1.77	2.18***	1.59–2.98
Grip strength in kg	0.95***	0.94–0.96	0.96***	0.94–0.97
Limitations in the Activities of Daily Living (yes/no)	2.88***	1.88–4.39	2.45***	1.64–3.68
Body Mass Index (BMI) in kg/m^2^	1.05***	1.03–1.08	1.05***	1.03–1.08
Interpersonal factors				
Marital Status				
Married	reference		reference	
Never married	1.92**	1.17–3.15	1.56	0.95–2.58
Divorced	0.94	0.62–1.44	0.65**	0.46–0.91
Widowed	2.07**	1.27–3.39	1.21	0.93–1.57
Household size	1.06	0.93–1.22	1.03	0.90–1.18
Partner in household (yes/no)	0.59***	0.44–0.79	0.93	0.76–1.14
Size of family network	0.94**	0.90–0.99	0.98	0.94–1.03
Number of grandchildren	0.96**	0.94–0.98	1.00	0.98–1.02
Size of social network^1^	0.88***	0.81–0.95	0.88**	0.81–0.95
Perceived social support^1^	1.44*	1.01–2.07	1.32	0.98–1.78
Perceived quality of social network^1^	0.97	0.89–1.05	0.98	0.90–1.06

* *p*-value< 0.05; ** *p*-value< 0.01; *** *p*-value< 0.001

^1^These variables are only available from wave 4 on in SHARE. Therefore, these were only used as an addition for the cross-sectional analyses to the interpersonal variables present in wave 1

Longitudinally, we found a protective effect on IPA for higher education (HR: 0.80; 95%-CI: 0.67–0.95) and grip strength (HR: 0.99; 95%-CI: 0.98–0.99) at baseline (Table 4). Whereas the number of chronic diseases (HR: 0.80; 95%-CI: 0.67–0.95), depression (HR: 1.31; 95%-CI: 1.10–1.56) and BMI (HR: 1.02; 95%-CI: 1.00–1.04) at baseline were statistically significant risk factors for IPA seven years later. In a fully adjusted model only grip strength (HR: 0.99; 95%-CI: 0.98–0.99) and BMI (HR: 1.03; 95%-CI: 1.01–1.05) remained statistically significant.

**Table 4:** Longitudinal intrapersonal factors of insufficient physical activity (IPA) at age 65–75 in prior active persons^1^

	Raw (*n* = 1515)		Adjusted (*n* = 1515)	
	Hazard ratio	95% confidence interval	Hazard ratio	95% confidence interval
Intrapersonal factors				
Age in years	1.01	0.99–1.03	–	–
Higher Education (yes/no)	0.80**	0.67–0.95	–	–
Books at the age of 10 years (yes/no)	0.95	0.81–1.11	–	–
Difficult financial situation of household (yes/no)	1.11	0.93–1.32	–	–
Number of chronic diseases	1.09**	1.03–1.16	–	–
Depression (yes/no)	1.31**	1.10–1.56	–	–
Hospitalization in the last 12 month (yes/no)	1.12	0.88–1.43	–	–
Grip strength in kg	0.99***	0.98–0.99	0.99***	0.98–0.99
Limitations in the Activities of Daily Living (yes/no)	1.10	0.75–1.62	–	–
Body Mass Index (BMI) in kg/m^2^	1.02*	1.00–1.04	1.03**	1.01–1.05

* *p*-value< 0.05; ** *p*-value< 0.01; *** *p*-value< 0.001; ^1^ prior = 7 years ago

For the interpersonal factors in the longitudinal analyses, it was found that the only factor showing an influence on IPA in previously active 65–75 year-olds was living together with the partner in one household at baseline (Table 5, HR: 0.74; 95%-CI: 0.56–0.97). This influence was only shown in men.

**Table 5:** Longitudinal interpersonal factors of insufficient physical activity (IPA) at age 65–75 in prior active persons^1^

	Male (*n* = 794)		Female (*n* = 721)	
	Hazard ratio (raw)	95% confidence interval	Hazard ratio (raw)	95% confidence interval
Interpersonal factors				
Marital Status				
Married	reference		reference	
Never married	0.69	0.43–1.11	1.00	0.85–1.19
Divorced	0.82	0.42–1.60	1.04	0.78–1.38
Widowed	0.88	0.47–1.67	0.93	0.70–1.23
Household size	0.93	0.81–1.07	1.05	0.92–1.19
Partner in household (yes/no)	0.74*	0.56–0.97	1.03	0.83–1.27
Size of family network	0.98	0.96–1.01	1.01	0.98–1.03
Number of grandchildren	0.99	0.97–1.02	1.02	0.99–1.05

* *p*-value< 0.05; ** *p*-value< 0.01; *** *p*-value< 0.001; ^1^ prior = 7 years ago

## Discussion

We investigated cross-sectional and longitudinal associations of a broad range of intra- and interpersonal factors with IPA.

### Cross-sectional associations

Regarding to the intrapersonal level, women were less physically active than men. These results are in line with previous research: A cross-sectional study using objective measurements of PA showed that 31% of older men and 20% of older women meet the WHO recommendations of 2.5 h moderate PA per week [50]; in questionnaire-based studies 16–21% in men and 11–21% in women were categorized as sufficiently physically active [15, 51]. In the SHARE data, we found an increasing proportion of IPA with age in both sexes. The influence of age on PA was confirmed in several previous studies [20–23]. Other intrapersonal factors of IPA that reached statistical significance were low educational level and financial difficulties. There is ample evidence for an association of socio-economic status with PA in the general population and this is as well true for older adults [20, 22]. Interestingly, we were also able to establish an association between parental education and IPA in older adults. Even in adulthood, the influence of parental education on several lifestyle factors was confirmed in studies [27, 28]. A previous study reported an association between childhood socioeconomic position and physical capability levels in adulthood [52]. Likewise, a number of health-related and fitness-related factors were associated to IPA. Many previous studies showed that good health in general is positively associated to PA in older adults [20, 22, 24, 25]. There are fewer studies on fitness-related factors in this age group. However, our findings of a positive association between higher grip strength and PA in older adults are consistent with a previous study [53].

The investigated interpersonal factors display several statistically significant associations with IPA, especially in men. The size of the social network as well as the size of the family network are both negatively associated with IPA, i.e. the larger these networks are the less likely it is to perform IPA. This is in line with previous research, which stated social networks and social support from friends and peers as influential factors for PA in older adults [20, 29]. However, we found that the perceived quality of the social network has no association with IPA. The perceived social support is even positively associated with IPA. One reason for this result might be that the SHARE data only includes received instrumental social support. Additional analyses showed that this result is rather a statistical artefact that can be fully explained by partner in household in men and by frailty status in women (data not shown). A recent Australian study showed that the association between social network and PA might be more complex and dependent on the type of activity [54], e.g. household activities and leisure-time activities. Distinctive gender patterns were present regarding family indicators, marital status, partner in household and number of grandchildren. In men, being married, presence of a partner in household and number of grandchildren showed all a statistically significant positive association with IPA. In women, the only interpersonal factor being statistically significant associated with IPA was being divorced. A clear gender difference of the health effects of divorce or being single was reported before [55].

In this study, the prevalence of IPA in 65–75-year-olds varied widely between countries, ranging from 55.4% to 83.3% in women and from 46.6% to 73.7% in men. A comparison of the IPA prevalence of the sub set used in this study and all participants of SHARE in this age group showed that most prevalence rates are in line with each other. Only the IPA prevalence for Germany, Austria and Denmark only in women differed (see Additional file 1). A German health survey showed an IPA prevalence in Germany of 60% in men and 63% in women in the age group of 60 to 69 years, which are higher values compared to those presented in this study [56]. For Austria and Denmark no prevalence rates of IPA where found for the age group 65 to 75 years. Therefore, the prevalence rates of the mentioned countries must be considered with caution. Women and men were more likely to meet the WHO recommendations of PA in northern and central European countries, as Sweden or Germany, and compared to southern European countries, as Italy and Spain. The reported increase in IPA from Northern and Central Europe to Southern Europe are in line with findings of a study of the European Commission in 2014 [14]. Climate, environmental, infrastructural and policy conditions for PA can differ highly within countries such as weather, topography, culture, street layout and crime rate. In our study, older adults from the Netherland, Germany, Denmark and Switzerland showed lower rates of overall IPA. A possible explanation is that these countries are all good practice examples for high levels of cycling and street safety [57], setting the ground for physical activity in older adults.

### Longitudinal analyses

Longitudinally, educational level, number of chronic diseases, presence of depression, grip strength and BMI were statistically significant predictors of IPA at the age of 65 to 75 in previously active persons. Of the investigated interpersonal factors, only living together with a partner in one household showed a statistically significant influence in men, but not in women.

In the multivariable model, only grip strength and BMI remained statistically significant predictors for IPA in older adults. Previous cross-sectional studies that were not able to distinguish between cause and effect showed that BMI is associated with PA in older adults [20, 23, 50, 58].

### Limitations and strengths

There are several limitations to the study. One limitation is the measurement of PA by questionnaire. Objective methods using accelerometers are more valid for PA measurements [59], but many large population studies did not use accelerometers due to high costs. In SHARE, only information on the frequency but not on the duration of moderate and vigorous PA is available. Thus, it can only be determined with some certainty that the WHO recommendations are not met, if persons engage in PA at most once a week. Moreover, there is a certain risk of social desirability bias due to the interview method (computer assisted personal interview) and the interviewee knowing that sufficient PA is desirable [60]. Previous studies have found that questionnaire-based surveys are likely to overestimate duration and intensity of PA compared to objective measurements [59]. A further limitation is the restricted set of variables that leaves out further putative interesting factors from investigation as e.g. social support as noted above or extrapersonal variables, which could help explaining cross-country differences, as built environment, weather conditions, cultural or policy factors [31, 36–39]. Finally, we restricted our analyses to a subset of the SHARE participants (participation in all 4 waves) to be able to analyse longitudinal effects. A questionnaire survey of non-responder in Germany showed only small indication for a non-response bias with regard to household composition and health status [43, 61]. Additionally, subgroup analyses indicated only small differences in sex and age in survey participation and retention [43, 53, 62].

The study also has several strengths. SHARE is conducted in several European countries with a highly standardized study protocol involving physical measurements as e.g. grip strength and BMI [43]. SHARE provides panel data of high quality and representativeness due to the high degree of standardization in data collection. It allows comparing several European countries and the sample size is sufficient to detect smaller effects and has high participation rates as well as only small evidence for attrition bias [53]. The longitudinal nature of the data enables identification of temporality of associations as e.g. BMI and physical activity [63].

## Conclusions

The detected variations in IPA in the northern and southern countries indicate an association between PA in older adults and extrapersonal factors. Therefore, further studies are needed, looking at extrapersonal factors in detail. Also, more studies are needed reflecting the complex nature of interpersonal factors in older adults on the one hand and PA on the other hand. We found a predominant influence of intrapersonal factors on meeting physical activity recommendations in older age for men and women. Longitudinally, grip strength and BMI were the influential factors for IPA emphasizing the importance of health as prerequisite to PA participation. Intervention programs promoting PA in older adults could focus on this fact by providing PA opportunities for all capability levels recognizing limitations that can typically occur at this age group.

## Additional file

Additional file 1: “Prevalence rates (PR) of insufficient physical activity (IPA) of all participants in the age group 65 to 75 years at wave 4”. This file shows the prevalence rates of all participants of SHARE in the age group 65–75 years at wave 4 as a table. (DOCX 24 kb)

## Abbreviations

ADL: Activities of daily living
ADLI: Activities of daily living index
BMI: Body mass index
CAPI: Computer assisted personal interviews
CI: Confidence intervals
EU: European Union
HR: Hazard ratio
IPA: Insufficient physical activity
ISCED: International Standard Classification of Education
PA: Physical activity
POR: Prevalence odds ratio
SHARE: Survey of Health, Ageing and Retirement in Europe; STROBE, Strengthening the Reporting of Observational Studies in Epidemiology.
