# Supplementary material for: Cross-sectional and longitudinal factors influencing physical activity of 65 to 75-year-olds: a pan European cohort study based on the survey of health, ageing and retirement in Europe (SHARE)
Source: BMC Geriatr. 2018 Apr 16;18:94. doi: 10.1186/s12877-018-0781-8 (PMC5902922; doi:10.1186/s12877-018-0781-8)
Supplement: Supplementary file 1 — “Prevalence rates (PR) of insufficient physical activity (IPA) of all participants in the age group 65 to 75 years at wave 4”. This file shows the prevalence rates of all participants of SHARE in the age group 65–75 years at wave 4 as a table. (DOCX 24 kb) [file 12877_2018_781_MOESM1_ESM.docx]

**Prevalence rates (PR) of insufficient physical activity (IPA) of all participants in the age group 65 to 75 years at wave 4**

| **Male** | | | | **Female** | | | |
| --- | --- | --- | --- | --- | --- | --- | --- |
|  | **Wave 1** baseline*  (n=4189)  PR | **Wave 2** 2 years later  (n=4002)  PR | **Wave 4** 7 years later  (n=5171)  PR |  | **Wave 1** baseline*  (n=4658)  PR | **Wave 2** 2 years later  (n=4430)  PR | **Wave 4** 7 years later  (n=5785)  PR |
| **Country** |  |  |  | ***Country*** |  |  |  |
| Sweden | (n=550)  49.8% | (n=505)  56.8% | (n=389)  47.6% | *Sweden* | (n=611)  62.2% | (n=576)  63.4% | (n=440)  65.9% |
| Denmark | (n=258)  48.8% | (n=408)  56.6% | (n=313)  60.1% | *Denmark* | (n=280)  56.1%** | (n=413)  65.9% | (n=303)  69.0% |
| Netherlands | (n=521)  51.2% | (n=428)  50.9% | (n=435)  52.4% | *Netherlands* | (n=517)  55.7% | (n=466)  45.9% | (n=461)  60.7% |
| Germany | (n=602)  51.8%** | (n=518)  56.4% | (n=337)  61.4%** | *Germany* | (n=606)  58.6%** | (n=489)  64.2% | (n=304)  57.2% |
| Belgium | (n=571)  62.0% | (n=450)  62.0% | (n=602)  64.3% | *Belgium* | (n=634)  75.7% | (n=513)  77.0% | (n=712)  80.6% |
| Austria | (n=286)  65.0% | (n=209)  65.1% | (n=747)  63.7%** | *Austria* | (n=356)  75.0% | (n=267)  67.8% | (n=964)  70.0%** |
| Switzerland | (n=164)  41.5% | (n=224)  43.3% | (n=554)  55.1% | *Switzerland* | (n=177)  55.4% | (n=266)  53.4% | (n=598)  65.4% |
| France | (n=408)  60.0% | (n=375)  65.9% | (n=669)  69.4% | *France* | (n=490)  74.7% | (n=487)  77.4% | (n=797)  81.1% |
| Italy | (n=488)  62.1% | (n=547)  68.9% | (n=614)  71.3% | *Italy* | (n=573)  72.1% | (n=597)  76.0% | (n=661)  82.8% |
| Spain | (n=341)  62.8% | (n=338)  63.6% | (n=511)  76.5% | *Spain* | (n=414)  71.0% | (n=356)  71.1% | (n=545)  81.7% |
| **Total** | **(n=4189)**  **56.1%** | **(n=4002)**  **59.4%** | **(n=5171)**  **63.2%** | ***Total*** | **(n=4658)**  **66.5%** | **(n=4430)**  **67.0%** | **(n=5785)**  **73.1%** |

*Age at baseline 58-68 years; **Deviation of more than 5 percentage points from prevalence rates presented in the results section of the study
